# Comparative Analysis of Spontaneous and Stimulus-Evoked Calcium Transients in Proliferating and Differentiating Human Midbrain-Derived Stem Cells

**DOI:** 10.1155/2017/9605432

**Published:** 2017-10-22

**Authors:** Torben Johansen, Christina Krabbe, Sissel Ida Schmidt, Alberto Martínez Serrano, Morten Meyer

**Affiliations:** ^1^Department of Cardiovascular and Renal Research, University of Southern Denmark, J.B.Winsløws Vej 21, 5000 Odense C, Denmark; ^2^Department of Neurobiology Research, Institute of Molecular Medicine, University of Southern Denmark, J.B.Winsløws Vej 21, 5000 Odense C, Denmark; ^3^Department of Molecular Biology and Center of Molecular Biology Severo Ochoa, UAM-CSIC, Campus Cantoblanco, 28049 Madrid, Spain

## Abstract

Spontaneous cytosolic calcium transients and oscillations have been reported in various tissues of nonhuman and human origin but not in human midbrain-derived stem cells. Using confocal microfluorimetry, we studied spontaneous calcium transients and calcium-regulating mechanisms in a human ventral mesencephalic stem cell line undergoing proliferation and neuronal differentiation. Spontaneous calcium transients were detected in a large fraction of both proliferating (>50%) and differentiating (>55%) cells. We provide evidence for the existence of intracellular calcium stores that respond to muscarinic activation of the cells, having sensitivity for ryanodine and thapsigargin possibly reflecting IP_3_ receptor activity and the presence of ryanodine receptors and calcium ATPase pumps. The observed calcium transient activity potentially supports the existence of a sodium-calcium antiporter and the existence of calcium influx induced by depletion of calcium stores. We conclude that the cells have developed the most important mechanisms governing cytosolic calcium homeostasis. This is the first comparative report of spontaneous calcium transients in proliferating and differentiating human midbrain-derived stem cells that provides evidence for the mechanisms that are likely to be involved. We propose that the observed spontaneous calcium transients may contribute to mechanisms involved in cell proliferation, phenotypic differentiation, and general cell maturation.

## 1. Introduction

Calcium is a versatile intracellular messenger controlling a wide range of cellular processes [1–3] including cell proliferation, cell differentiation, and general gene transcription [4–7]. Calcium signals are considered to be involved in fertilization of most species [8–11] as well as in the subsequent embryonic development [12–18].

Spontaneous calcium transients and oscillations have been reported in a number of tissues of nonhuman origin [19]. More recently, spontaneous calcium oscillations have been observed in early postnatal cerebellar Purkinje neurons [20], embryonic mouse cortical brain slices [21], mouse spinal cord neurons [22], slice cultures of the spinal cord and dorsal root ganglia prepared from mouse embryos [23], and undifferentiated cells and neural progenitor cells derived from a mouse bone marrow [24]. There have also been reports on spontaneous calcium oscillations in human mesenchymal stem cells [25–27], human embryonic stem cell-derived neurons [28], and human cardiac progenitor cells [29]. It appeared that calcium supply to cytosol was derived from intracellular calcium stores by IP_3_-dependent release and influx of calcium through store-operated channels. Removal of calcium was dependent on plasma membrane calcium pump activity and Na^+^-Ca^2+^ exchange [25–27].

It has been reported that the mechanisms that regulate spontaneous calcium oscillations in stem cells may change during their transition from proliferation to differentiation and maturation [27, 30–33]. However, no reports describe changes in calcium regulation specifically in human midbrain-derived stem cells during their development to neurons.

The present investigation was initially performed in order to study spontaneous calcium signaling in human midbrain-derived stem cells undergoing neuronal differentiation [34–37]. Since we observed spontaneous calcium transients in a large fraction of the differentiating cells, it was of interest to further explore the development of calcium-regulating mechanisms by comparing the mechanisms in differentiating cells with mechanisms operating in proliferating cells.

Using a confocal imaging technique, we here provide evidence for the existence of intracellular calcium stores that respond to muscarinic activation, having sensitivity to ryanodine and thapsigargin possibly reflecting IP_3_ receptor activity and the presence of ryanodine receptors and calcium ATPase pumps. The observed calcium transient activity may support the existence of a sodium-calcium antiporter and the existence of calcium influx induced by depletion of calcium stores.

We conclude that identical calcium-regulating mechanisms operate during the proliferation and neuronal differentiation of human midbrain-derived stem cells.

## 2. Materials and Methods

### 2.1. Ethics Statement

Human tissues were donated for research after written informed consent of women seeking abortion was provided. Tissue procurement was performed in accordance with the Declaration of Helsinki and in agreement with the ethical guidelines of the Network of European CNS Transplantation and Restoration (NECTAR). Approval to use these tissues for research was granted by the Lund University Hospital Ethical Committee, and their use was in compliance with Spanish law 35/1988 on Assisted Reproduction. Ethics statements about the human fetal origin of the cells used in the present study can be found in the original report describing the cell line [37].

### 2.2. Culturing and Passaging of Human Midbrain-Derived Stem Cells

Cell isolation, genetic modifications, and general characterizations are described elsewhere [34–37]. Briefly, cells were isolated from the ventral mesencephalon of a 10-week-old human fetus. Immortalization was carried out by infection with a retroviral vector coding for v-myc. Derivatives of the resulting cell line (hVM1 cells) were used for stable retroviral overexpression of Bcl-X_L_, essentially as described by Liste et al. [38]. After infection, the cells were selected by GFP-based fluorescent-activated cell sorting, resulting in the cell line hVMbcl-x_L_ [34–36] used in the present study.

Cells at passage 20 were plated onto poly-L-lysine- (PLL; Sigma-Aldrich, St. Louis, MO, USA) coated T75 culture flasks containing a 15 ml HNSC100 culture medium (DMEM/F12 with Glutamax (Gibco, Rockville, MD, USA), 0.6% (*w*/*v*) d-glucose (Sigma-Aldrich), 0.5% (*v*/*v*) 1 M Hepes (Gibco), 0.5% (*w*/*v*) AlbuMAX-I (Gibco), 1% (*v*/*v*) N2 supplement (Gibco), 1% (*v*/*v*) NEAA (Sigma-Aldrich), and 1% Pen/Strep (Gibco)) supplemented with 20 ng/ml recombinant human epidermal growth factor (EGF, R&D Systems) and 20 ng/ml recombinant human basic fibroblast growth factor (bFGF, R&D Systems). Cells were propagated at 36°C in a controlled atmosphere of 5% CO_2_ and 95% humidified air. The medium was changed every third day, and cells passaged at 80% confluence. For passaging, adherent cells were washed with phosphate buffered saline (PBS) without calcium and magnesium, before detachment by trypsin-EDTA (Gibco) diluted 1 : 10 in PBS for 3–5 min at 36°C. Trypsinized cells were resuspended, centrifuged for 5 min at 4°C (130*g*), and plated in new flasks.

### 2.3. Neuronal Differentiation Protocol

Stem cells were differentiated into neurons (>90%) using the CK4 protocol [34, 35]. In brief, cells were plated in PLL-coated 24-well culture trays (Nunc) at a density of 5000 cells/cm^2^ in a HNSC100 medium with sequential addition of 50 ng/ml recombinant fibroblast growth factor 8 (R&D Systems), 25 *μ*M forskolin (Sigma-Aldrich), 5 ng/ml recombinant glial cell line-derived neurotrophic factor (Promega, Madison, WI, USA), and 25 ng/ml recombinant sonic hedgehog (R&D Systems). Cells for fluorescence staining were grown on 14 mm PLL-coated glass coverslips. Cells were differentiated for 10 days at 36°C in controlled atmosphere of 5% CO_2_ and 95% humidified air, with half of the medium being changed every third day.

### 2.4. Cell Fixation and Immunocytochemistry

Cells were fixed in 4% paraformaldehyde in 0.15 M PBS, pH 7.4 for 20 min and then rinsed for 3 × 15 min in 0.05 M Tris buffered saline (TBS, pH 7.4)/0.1% Triton X-100 (Sigma-Aldrich). Cultures were then preincubated for 30 min in TBS/10% donkey (Millipore) or sheep serum (Sigma-Aldrich) before incubation with one of the following primary antibodies in TBS/10% donkey or sheep serum for 24 h at 4°C: beta-tubulin III (*β*-tub III, monoclonal mouse; Sigma-Aldrich) 1 : 2000; tyrosine hydroxylase (TH, polyclonal rabbit; Chemicon) 1 : 1200; *γ*-aminobutyric acid (GABA, polyclonal anti-rabbit; Sigma-Aldrich) 1 : 500; microtubule-associated protein 2ab (Map2, monoclonal anti-mouse; Sigma-Aldrich) 1 : 2000; glial fibrillary acidic protein (GFAP, polyclonal anti-rabbit; DAKO, Carpenteria, CA, USA) 1 : 5000; doublecortin (DCX, polyclonal guinea anti-pig; Chemicon) 1 : 400; human nuclei (HN, monoclonal anti-mouse; Chemicon) 1 : 500; nestin (anti-human; AbD Serotec) 1 : 2000; and Ki67 (polyclonal anti-rabbit; Neomarkers Inc.) 1 : 500. Control stainings were performed by excluding primary antibodies or using IgG rabbit (DAKO) 1 : 20,000 and IgG_1_ mouse (DAKO) 1 : 200.

Cultures were rinsed for 3 × 15 min in TBS/0.1% Triton-X-100 and then incubated for 1 h with one of the following biotinylated secondary antibodies in TBS/10% donkey or sheep serum: anti-guinea pig Ig (Jackson ImmunoReseach) 1 : 200, anti-rabbit Ig 1 : 200 or anti-mouse Ig 1 : 200 (Amersham). Cells were rinsed for 3 × 15 min in TBS/0.1% Triton X-100 and incubated for 60 min with horseradish peroxidase- (HRP-) conjugated streptavidin (DAKO) diluted 1 : 200 in TBS/10% donkey or sheep serum. Cultures were then rinsed for 3 × 15 min in TBS before visualization with 3.3′-diaminobenzidine (50 mg 3.3′-diaminobenzidine (Sigma-Aldrich) and 33 *μ*l H_2_O_2_ per 100 ml TBS for 5–10 min). After rinsing for 15 min in TBS and briefly in distilled water, cultures were coverslipped in Aquatex (VWR).

Immunofluorescence staining of proliferating cell cultures was also performed. In brief, cultures were rinsed for 3 × 15 min in TBS/0.1% Triton X-100 and then preincubated for 30 min in TBS/5% goat serum (Chemicon) and then incubated for 24 h at 4°C with the following primary antibodies diluted in TBS/5% goat serum: HN (monoclonal anti-mouse; Chemicon) 1 : 500; nestin (polyclonal anti-rabbit; Abcam) 1 : 1000; and *β*-tub III (monoclonal anti-mouse; Sigma-Aldrich) 1 : 2000; Ki67 (polyclonal anti-rabbit; Neomarkers Inc.) 1 : 500. Subsequently, cells were washed for 3 × 15 min in TBS/0.1% Triton X-100 and incubated with Alexa Flour®555-conjugated mouse IgG and Alexa®488 anti-rabbit IgG for two hours. Plates were washed for 2 × 15 min in TBS and 1 × 15 min in TBS added 4′-6-diamidino-2-phenylindole (DAPI; Sigma-Aldrich) for general staining of cell nuclei. After a brief wash in distilled water, cells on coverslips were mounted with ProLong Gold (Molecular Probes, Grand Island, NY, USA) onto glass slides. Confocal pictures were taken using FluoView FV1000MPE -Multiphoton Laser Scanning Microscope (Olympus, Hamburg, Germany).

### 2.5. Measurements of Intracellular Calcium

Monolayers of stem cells proliferated for 2 days or differentiated for 10 days on PLL-coated glass coverslips were loaded with 5 *μ*M Fura-2/AM (acetoxymethyl ester of fura-2; Molecular Probes) or Fluo-4-AM (see below) for 30 min at room temperature in the dark. Extracellular dye was removed by repeated dilution of the sample in Krebs-Ringer solution. Then, the cells were incubated in a buffered salt solution appropriate for the experiments, that is, containing various concentrations of calcium or calcium free with or without EGTA added. For some experiments, addition of 2 *μ*M thapsigargin (Molecular Probes) to the calcium-free loading solution was used in order to deplete intracellular calcium stores.

The intracellular Ca^2+^ concentration was monitored using confocal microscopy (inverted epifluorescence microscope; Zeiss Axiovert 35) and a Ca^2+^ image system (TILL Photonics GmbH). Fura-2 was excited at 360 nm and 380 nm. Emission was measured at 510 nm through a long pass filter. Images were collected at a frame rate of 0.33 Hz. TILLVision software (TILL Photonics GmbH) was used to generate ratio images and to analyze individual cell fluorescence intensity by use of region of interest (ROI). Intracellular calcium ([Ca^2+^]_i_) is expressed as a ratio of fluorescence emission caused by excitation at 360 nm and 380 nm (excitation ratio 360/380 (F_360_/F_380_)). The performance of the image system was tested by external calibration using a calibration kit (Molecular Probes) (free Ca^2+^ concentration from 0 *μ*M to 39 *μ*M). The average isosbestic point was 363 nm, and we found an almost linear relation between [Ca^2+^] and the fluorescence intensity (excitation ratio 360/380) from 17 nM to 1.35 *μ*M of [Ca^2+^]. Fura-2-loaded cells were used to visualize the spontaneous transients in differentiated stem cells. Intracellular calcium in proliferating stem cells was measured using Fluo-4-loaded cells. Fluorescence measurements were performed using a FluoView FV1000MPE multiphoton laser scanning microscope (Olympus). Fluo-4 was excited at 488 nm. Emission was measured at 510 nm through a long pass filter. Images were collected at a rate of 0.45 Hz.

### 2.6. Chemicals

Fura-2-AM, Fluo-4-AM, thapsigargin, and Ryanodine Calibration kit were supplied by Molecular Probes. All other chemicals were purchased from Sigma-Aldrich and Merck.

Krebs-Ringer solution (in mM): NaCl, 139; KCl, 5; MgCl_2_, 1.2; CaCl_2_, 2; glucose, 10; HEPES, 10 pH was adjusted to 7.4 by NaOH. N-methyl-D-glucosamine (NMDG) replaced sodium in sodium-free solutions.

### 2.7. Data Analysis and Statistics

Data are reported as mean and SEM. Student's *t*-test was used for statistical comparisons of unpaired groups. A *p* value lower than 0.05 was considered statistically significant.

## 3. Results

### 3.1. General Immunocytochemical Characterization of Proliferating Stem Cells and Differentiating Derivatives

To study spontaneous calcium transients in human neurons, we used a well-characterized human ventral mesencephalic stem cell line [34, 35, 37]_._ Prior to analysis of calcium transients, cells were differentiated for 10 days *in vitro* according to our neuronal differentiation protocol (CK4 protocol) in which fibroblast growth factor 8 (50 ng/ml), forskolin (25 *μ*M), glial cell line-derived neurotrophic factor (5 ng/ml), and sonic hedgehog (25 ng/ml) are added in a sequential manner [34, 35].

**Figure 1:**
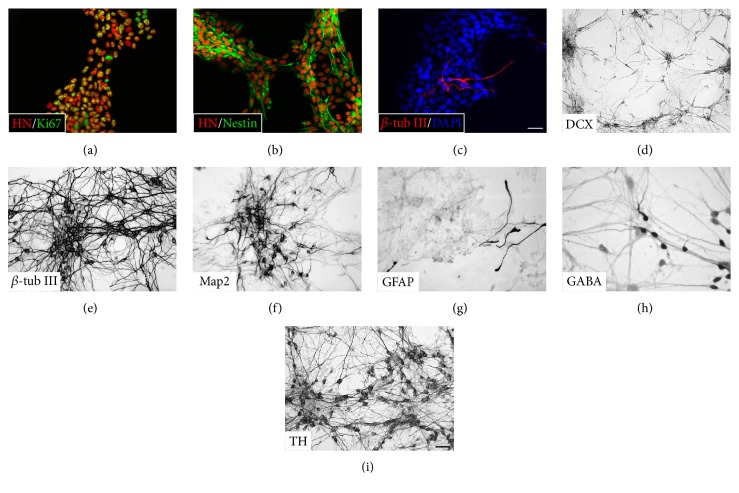
Immunocytochemical characterization of proliferating (a, b, c) and differentiating human midbrain-derived stem cells (d, e, f, g, h, i). Immunofluorescence staining of cultures propagated for 4 days by exposure to epidermal growth factor and basic fibroblast growth factor was performed. Almost all cells expressed Ki67, a marker of dividing cells (a). Moreover, a large proportion of the cells expressed the intermediate filament protein nestin, a marker of neural progenitor cells (b). Only very few cells had spontaneously differentiated into *β*-tubulin III- (*β*-tub III-) positive neurons (c). Cells were differentiated for 10 days by exposure to fibroblast growth factor 8 for three days followed by exposure to forskolin, sonic hedgehog, and glial cell line-derived neurotrophic factor for seven days and immunostained for neuronal and astroglial markers. At this time point, some cells expressed the early marker of migrating neuronal cell doublecortin (DCX) (d), whereas extensive staining for another early neuronal marker, *β*-tub III (e), and a mature neuronal marker, microtubule-associated protein 2ab (Map2) (f), was seen. A large proportion of cells expressed tyrosine hydroxylase (TH), a marker of catecholaminergic neurons (i), whereas only few cells were found to express *γ*-aminobutyric acid (GABA), a marker for GABAergic neurons (h). Very few cells were found to express glial fibrillary acidic protein (GFAP), a marker of astroglial cells (g). Scale bars = 50 *μ*m.

For initial general cytological characterization and quality control, cells were either propagated in the presence of epidermal growth factor (EGF, 20 ng/ml) and basic fibroblast growth factor (bFGF, 20 ng/ml) for 4 days or differentiated for 10 days as described above. Cells were then immunostained for markers of dividing cells, neural progenitor cells, newly generated neurons, mature neurons, astroglial cells, and specific neuronal subtypes (Figure 1).

To investigate the proliferative capacity in propagating cultures, cells were immunostained for Ki67. At day 4 of propagation, almost all cells were in different phases of cell division (Figure 1(a)), and they expressed the intermediate filament nestin (Figure 1(b)), which is a general marker of neural precursor cells. In propagating cultures, only few cells had started to differentiate into neurons as shown by the presence of very few *β*-tubulin III- (*β*-tub III-) positive cells, a marker of newly generated neurons (Figure 1(c)).

In 10-day-old differentiating cultures, several cells were found to express doublecortin (DCX) (Figure 1(d)), a microtubule-associated protein expressed in migrating and developing neurons that is downregulated in fully differentiated cells. Numerous *β*-tub III-positive neurons (Figure 1(e)) and microtubule-associated protein 2- (MAP2-) positive mature neurons were found in all differentiating cultures (Figure 1(f)). In contrast, only few cells stained positive for the astroglial marker glial fibrillary acidic protein (GFAP) (Figure 1(g)). Cells were also immunostained for markers of more specific neuronal subtypes. Numerous cells were found to express tyrosine hydroxylase (TH), a marker of catecholaminergic neurons (Figure 1(i)), whereas only few cells expressed a marker of GABAergic neurons (GABA) (Figure 1(h)).

Taken together, all stem cell cultures were viable and displayed a healthy appearance, and after differentiation, they were highly rich in neuronal cells (>90% of total cells).

### 3.2. Differentiating Midbrain-Derived Stem Cells

#### 3.2.1. Calcium Supply to Spontaneous Calcium Transients in Cytosol of Differentiating Neurons

**Figure 2:**
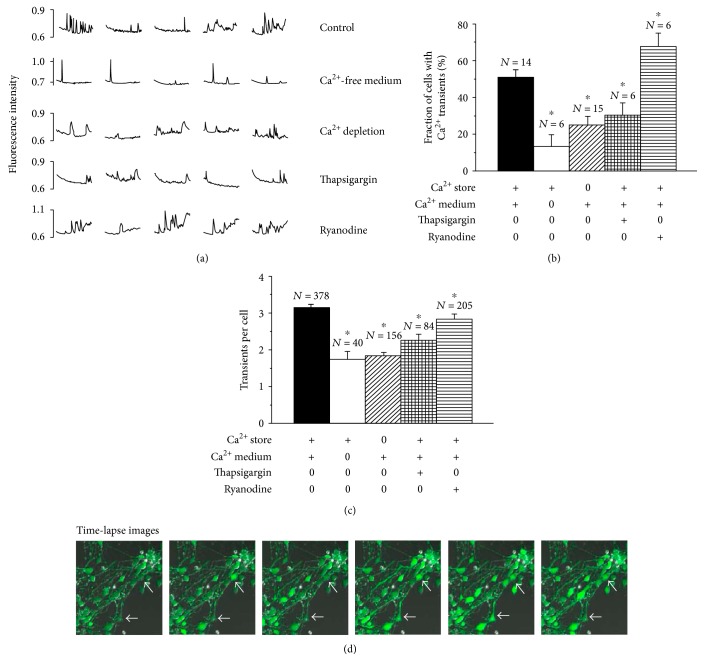
Spontaneous calcium transients in cytosol of differentiating stem cells are dependent on intracellular and extracellular calcium supply. Fura-2-loaded cells were measured for 20 min at room temperature. The pattern of calcium transients from five representative cells is shown (a). The fraction of cells with calcium transients (cytosolic calcium increase followed by decrease) was calculated in each experiment (b), as was the number of transients per cell (c). The cells were incubated in the presence or absence of extracellular calcium as indicated on the figure. Pretreatment with thapsigargin in the absence of calcium was used to deplete the intracellular calcium stores (third bars in the two histograms). Ryanodine was used to study the role of ryanodine receptors. The mean values and SEM from 6–15 independent experiments (b) and 40–378 cells (c) are shown. ∗ indicate significant difference from the control value. Consecutive recordings (still images) of Fluo-4-loaded cells with a 60 sec interval ((d); corresponding video available, see Supplemental Figure 1).

Spontaneous calcium transients were observed in 51% of the differentiating cells (Figures 2(a), 2(b), and 2(d); Supplemental Figure 1, video available online at https://doi.org/10.1155/2017/9605432). Removal of extracellular calcium as well as depletion of the intracellular calcium stores significantly reduced the fraction of cell with spontaneous calcium transients (*p* < 0.05). Depletion of the intracellular calcium stores was performed by exposure of the cells to 2 *μ*M thapsigargin in a calcium-free medium for 30 min before the experiments. The fraction of cells with spontaneous calcium transients was also significantly reduced by addition of 2 *μ*M thapsigargin to the cells even in the presence of extracellular calcium (*p* < 0.05). In contrast, incubation of the cells in the presence of 10 *μ*M ryanodine that binds to the ryanodine receptor in the endoplasmic reticulum [39, 40], significantly increased (*p* < 0.05) the fraction of cells with calcium transients. There were no calcium transients in store-depleted cells when incubated in a calcium-free medium (three independent control experiments, not shown).

The number of calcium transients during 20 min incubation in each differentiating cell was calculated (Figure 2(c)). A calcium transient was defined as a peak of 0.05 fluorescence units or more over baseline. We observed 3.38 (mean value) transients in the fraction of cells showing spontaneous calcium transients and intact calcium stores, when the cells were incubated in a calcium-containing medium. The number of transients per cell was significantly reduced if the cells were incubated in a calcium-free medium (1.88 transients per cell) as well as if the cellular calcium stores were depleted by preincubation of the cells with 2 *μ*M thapsigargin, even when calcium was present during the incubation period (1.98 transients per cell) (*p* < 0.05). When 2 *μ*M thapsigargin was added to the cells, even in presence of extracellular calcium, the number of transients per cell was also reduced (2.43 transients per cell) (*p* < 0.05). However, by incubation of the cells with 10 *μ*M ryanodine, the number of transients per cell (3.04 transients) was similar to the value observed for cells with intact calcium stores when incubated in a calcium-containing medium.

#### 3.2.2. Pattern of Cytosolic Calcium Transients in Differentiating Neurons

While the appearance of the spontaneous transients (Figure 2(a)) varied a lot in frequency and peak size in cells with intact calcium stores and exposed to extracellular calcium, only a few narrow peaks appeared regularly when extracellular calcium was omitted. The appearance of small but broad peaks was the result of calcium depletion. The resting level of cytosolic calcium between the peaks was not changed in these cells. In contrast, baseline calcium decreased gradually with time in thapsigargin-treated cells and increased gradually in ryanodine-treated cells. The transients tended to appear at the end of the observation period in thapsigargin-treated cells. In ryanodine-treated cells, there were many transients of various sizes.

#### 3.2.3. Effect of Thapsigargin on Cytosolic Calcium in Differentiating Neurons and Effect of Calcium Addition on Thapsigargin-Primed Cells

**Figure 3:**
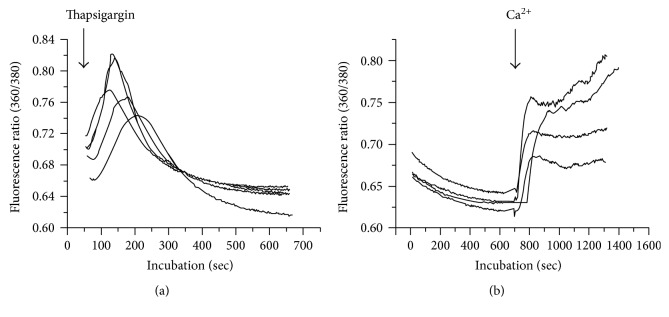
Effects of thapsigargin on cytosolic calcium (a) and calcium addition to store-depleted differentiating stem cells (b). Cells were incubated in a calcium-free medium with 10 *μ*M EGTA and then 2 *μ*M thapsigargin was added (arrow). The cells were dye loaded in Krebs-Ringer solution (containing 2 mM calcium). The results from five independent experiments are shown, each represents the mean value from 29 to 65 cells, all of which with a calcium transient in response to thapsigargin (a). The cells were dye loaded in the presence of calcium and thapsigargin (2 *μ*M). The dye was removed and the cells were primed with 2 *μ*M thapsigargin for 12 min in calcium-free solution. Then 2 mM calcium was added, and the incubation lasted for 8–11 min. The results from four independent experiments are shown, each representing the mean value obtained from analysis of 23 to 39 cells, all of which displayed calcium transients in response to the addition of calcium (b).

Exposure of the differentiating cells to 2 *μ*M thapsigargin induced a transient increase of cytosolic calcium in all the cells assessed in the five independent experiments (Figure 3(a)). The cells were incubated in calcium-free Krebs-Ringer solution containing 10 *μ*M EGTA to assure that no extracellular calcium would contribute to changes in cytosolic calcium.

The initial ratio had a mean value of 0.69 and was increased to a maximum mean value of 0.77 1-2 min after addition of thapsigargin. Then, it gradually decreased to a steady state level of a mean value of 0.65 during the following 5-6 min.

Figure 3(b) shows the effect of extracellular calcium on thapsigargin-primed differentiating neurons. There was a gradual decrease of the ratio from mean 0.67 to mean 0.63 during the initial incubation of the differentiating cells with thapsigargin in the absence of extracellular calcium. While the cells were incubated with thapsigargin, addition of calcium caused an immediate increase of the ratio to mean 0.73 within 2 min. The increased calcium was either unchanged or moderately elevated during the last 8–11 min of incubation (Figure 3(b)).

#### 3.2.4. Calcium-Induced Increase of Cytosolic Calcium in Differentiating Neurons Incubated in the Absence or Presence of Sodium

**Figure 4:**
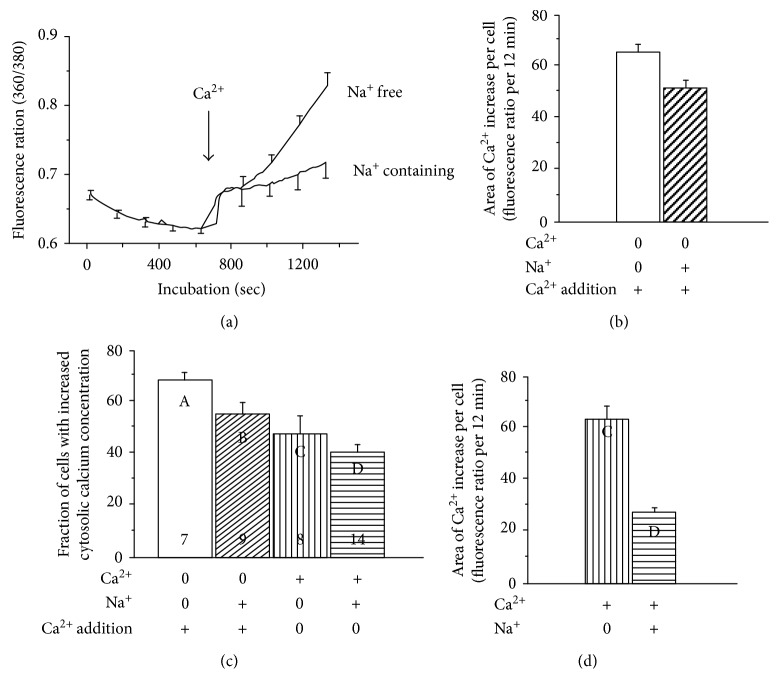
Calcium-induced increase of cytosolic Ca^2+^ in differentiating cells incubated in the absence or presence of Na^+^. After dye loading in the presence of Ca^2+^, cytosolic Ca^2+^ was monitored in cells incubated in a calcium-free medium with or without Na^+^ and then 2 mM Ca^2+^ was added (arrow) (a). The area under the curve (AUC) of the effect of Ca^2+^ addition is shown (b). The fraction of cells that respond to the addition of 2 mM Ca^2+^ with increased cytosolic Ca^2+^ is shown (c). The cells were incubated in the absence and presence of Na^+^ and then exposed to 2 mM Ca^2+^ ((c), columns A, B). Columns C and D show the fraction of cells with spontaneous Ca^2+^ increase in the absence and presence of Na^+^. AUC of the spontaneous Ca^2+^ increase is shown in columns C and D in Figure 4(d). GraphPad Prism4 was used to calculate AUC, expressed as fluorescence intensity (excitation ratio 360 nm/380 nm) per cell per 12 min (b) and 20 min (d), respectively. Mean value and SEM from 7 and 9 independent experiments (Figure 4(a)). Mean values and SEM from 296 cells and 270 cells incubated in the absence or presence of sodium, respectively (Figure 4(b)). Mean values and SEM from 7–14 experiments (Figure 4(c)) and 226 cells and 384 cells in Figure 4(d) (columns C and D, resp.). Column D in Figure 4(c) represents the data shown in Figure 2.

The differentiating cells were dye loaded in a calcium-containing solution. The extracellular dye was removed by repeated washing of the cells. They were then incubated for 10 min in a calcium-free medium in the absence or presence of sodium (Figure 4(a)). Addition of calcium caused a time-dependent, continuous increase of cytosolic calcium in cells incubated in sodium-free solution. In contrast, following an initial calcium increase, the cytosolic calcium concentration seemed to level off in cells incubated in the presence of sodium.

AUC (area under the curve) of the time-dependent changes in fluorescence intensity (excitation ratio 360 nm/380 nm) was significantly larger during calcium stimulation (12 min) when sodium was omitted from the incubation solution (Figure 4(b)) (*p* < 0.05). The fraction of cells with increased cytosolic calcium was calculated in each experiment (Figure 4(c)). Increased calcium in response to addition of calcium was observed in 86% and 70% of cells in the absence (A) or presence (B) of sodium, respectively (*p* < 0.05). In contrast, the spontaneous calcium increase in cytosol was not influenced by sodium (C, D). However, omission of sodium greatly enhanced the cytosolic calcium concentration expressed as AUC during the spontaneous increase of cytosolic calcium (column C in Figure 4(d)) when compared to spontaneous calcium transients in cells incubated in sodium containing Krebs-Ringer solution (*p* < 0.0001) (column D in Figure 4(d)).

#### 3.2.5. Effect of Carbachol on Differentiating Neurons

Expression of genes encoding the cholinergic muscarinic receptors (CHRM_1–5_) was confirmed using gene expression microarrays (data not shown).

Carbachol is a stable muscarinic agonist used to study the response of the differentiating stem cells to muscarinic Ach receptor activation [41]. There was an almost linear relation between the log dose of carbachol and the fraction of cells with calcium transients in each experiment (Supplemental Figure 2A). The cells were exposed to carbachol for 15 min. Omission of extracellular calcium had no effect on the cellular response. However, the response was inhibited by pirenzepin (10 *μ*M), a muscarinic M_1_ receptor antagonist [40]. In order to quantify the intracellular calcium increase, we calculated AUC by use of GraphPad Prism 4 software. The baseline for the calculation was estimated by use of Origin 7.5. The AUC is expressed in arbitrary units as fluorescence intensity per 15 min. Supplemental Figure 2B shows a dose-response curve of the effect of carbachol on the mean value of AUC per cell of all the active cells. 10 *μ*M and 100 *μ*M carbachol caused a large increase of AUC, which was counteracted by 10 *μ*M pirenzepine. In calcium-free solution, there was almost no effect of carbachol.

### 3.3. Proliferating Midbrain-Derived Human Stem Cells

**Figure 5:**
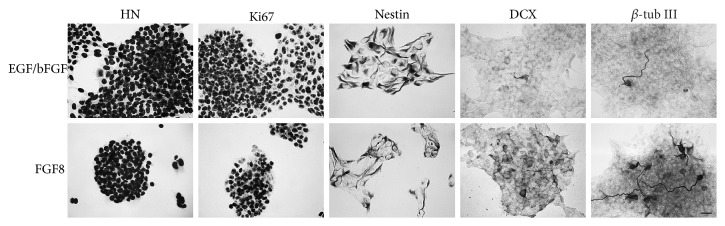
Immunocytochemical characterization of proliferating human midbrain-derived stem cells. Immunostaining of cells during, (1) standard propagation (upper panel) in a medium with epidermal growth factor (EGF) and basic fibroblast growth factor (bFGF) and (2) after removal of EGF/bFGF and one-day exposure to fibroblast growth factor 8 (FGF8) (lower panel), which represents the initial step of the induced neuronal differentiation (sequential addition of various factors, see Materials and Methods for details). To visualize all cells, cultures were immunostained using an antibody against human nuclei (HN). Almost all cells in both experimental groups expressed Ki67, a marker of dividing cells. Moreover, a very large proportion of the cells expressed the intermediate filament protein nestin, a marker of neural progenitor cells. Only very few cells had spontaneously differentiated into *β*-tubulin III- (*β*-tub III-) positive or doublecortin- (DCX-) positive immature neurons in the EGF/bFGF group, whereas some *β*-tub III-positive and DCX-positive neuronal cells were seen after short-term FGF8 treatment. Scale bar = 20 *μ*m.

With the aim to investigate spontaneous calcium transients and calcium-regulating mechanisms, our analyses were extended to include proliferating stem cells. As mentioned in the general immunocytochemical characterization and shown in Figures 1 and 5, the immature stem cells expressed the proliferation marker Ki67 and the precursor cell marker nestin. Only few cells (less than 0.1%) displayed spontaneous differentiation and expressed *β*-tub III.

#### 3.3.1. Effect of Thapsigargin and Calcium on Proliferating Stem Cells Incubated in a Calcium-Free Medium

Exposure of the cells to 2 *μ*M thapsigargin in a calcium-free medium induced a transient increase of cytosolic calcium in more than 2/3 of the cells in two independent experiments (Supplemental Figure 3). The cells were incubated in a calcium-free Krebs-Ringer solution containing 10 *μ*M EGTA to assure that no extracellular calcium would contribute to changes in cytosolic calcium.

Stem cells were also primed with 2 *μ*M thapsigargin for 30 min in a calcium-free Krebs-Ringer solution. Addition of 2 mM calcium (without thapsigargin) induced a calcium transient in seven independent experiments containing 332 cells that all responded with a calcium transient to the addition of calcium (Supplemental Figure 4). These experiments are thus similar to the experiments illustrated in Figure 3(a).

**Figure 6:**
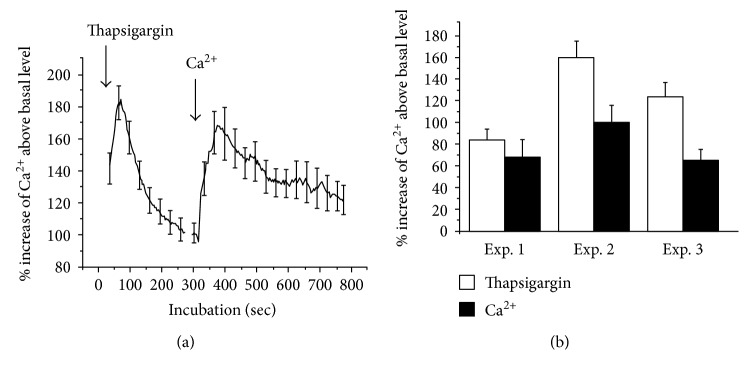
Effect of thapsigargin and calcium on proliferating stem cells incubated in a calcium-free medium. The cells were dye loaded and washed in a calcium-containing medium and then incubated in a calcium-free medium with 10 *μ*M EGTA. Thapsigargin (2 *μ*M, final concentration) was added after initiation of the incubation. After incubation for 300 sec, 2 mM calcium (final concentration) was added to the cells. The results from one of three independent experiments are shown (a). Abscissa: time of incubation (sec). Ordinate: intracellular calcium content. The fluorescence intensity was normalized to the level of the fluorescence intensity of untreated cells (100%). Mean values and SEM from 31 cells. All the cells responded to the addition of thapsigargin and calcium. Peak values from all three experiments, after addition of thapsigargin (open column) and calcium (closed column) can be seen (b). Mean values and SEM from 31–64 cells. Taken together, 149 cells were studied in three independent experiments, and 98% of the cells responded to the addition of both thapsigargin and calcium.

When the cells were loaded in the presence of calcium and incubated in a calcium-free medium with the addition of thapsigargin followed by the addition of calcium, two calcium transients were observed (Figure 6).

#### 3.3.2. Spontaneous Calcium Transients in Proliferating Stem Cells

**Figure 7:**
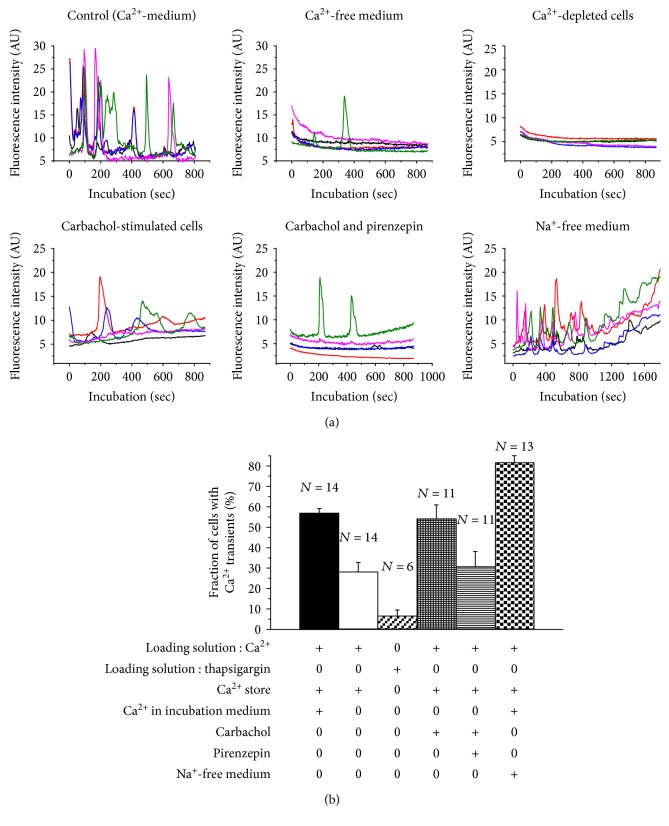
The fraction of proliferating cells with calcium transients during 15 min of incubation. Representative calcium traces from cells kept under six different experimental conditions (trace from five cells in each group) (a). The cells were loaded with 5 *μ*M Fluo-4. Calcium was present during the loading and the incubation procedures as indicated on the figure. Thapsigargin was used for depletion of calcium stores. Thapsigargin was present during the loading procedure. Carbachol and pirenzepin were present during the incubation. Sodium-free solution was used only for the incubation. Mean values and SEM, 6–14 independent experiments (b).

Representative calcium traces from cells were kept under six different experimental conditions (traces from five cells in each group) (Figure 7(a)). Spontaneous calcium transients were observed in 57% of cells incubated in Krebs-Ringer solution with 2 mM calcium (Figure 7(b); control sample; 1st bar). Omission of calcium in the incubation medium (Figure 7(b); 2nd bar) caused a significant decrease of the fraction of cells with calcium transients to 28% (*p* < 0.001). Depletion of intracellular calcium by loading of cells with Fluo-4 in the presence of 2 *μ*M thapsigargin in a calcium-free medium followed by incubation in a calcium-free medium further decreased the fraction of cells with calcium transients to 7% (*p* < 0.05, compared to samples in calcium-free solution) (Figure 7(b); 3rd bar).

Addition of 10 *μ*M carbachol to the incubation medium increased the fraction of cells with calcium transients to 54%, and this was counteracted by adding 10 *μ*M pirenzepine to the incubation medium (*p* < 0.05; 4th versus 5th bar) (Figure 7(b)).

Incubation of cells in a sodium-free salt solution with calcium (Figure 7(b); 6th bar) significantly increased the fraction of cells with calcium transients to 81% (*p* < 0.001, compared to control value). Sodium was replaced by NMDG in order to maintain physiological osmolality.

#### 3.3.3. Pattern of Cytosolic Calcium Transients in Proliferating Stem Cells

The appearance of the traces of control samples, samples from cells in a calcium-free medium as well as calcium-depleted cells, was similar to the trace observed with differentiated cells. Carbacholine-stimulated cells showed broad calcium transients, and the baseline calcium level gradually increased during the incubation in a sodium-free medium.

## 4. Discussion

### 4.1. Differentiating Human Midbrain-Derived Stem Cells

Calcium signaling has essential roles in the development of the nervous system from neural induction to the proliferation, migration, and differentiation of neural cells [42-44].

We have observed spontaneous elevations of intracellular calcium concentrations (calcium transients) in human midbrain-derived stem cells undergoing neuronal differentiation (see Supplemental Figure 1 (video) and Figure 2). The calcium transients varied in rate and shape. The basal calcium level seemed to change according to the use of pump and calcium channel-active drugs used for the experiments (thapsigargin and ryanodine, resp.). The source of calcium for the calcium transients might be the extracellular calcium which enters the cytosol through calcium channels or intracellular calcium stores releasing calcium by opening IP_3_- or ryanodine-sensitive channels.

#### 4.1.1. Calcium Source to Spontaneous Calcium Transients in Differentiating Neurons

Removal of extracellular calcium greatly reduced the fraction of cells in each experiment with calcium transients demonstrating that the opening of plasma membrane calcium channels contributes to the calcium transients. Similar results were previously reported for human embryonic stem cell-derived neurons [28]. The number of transients per active cell decreased in the absence of external calcium but the remaining activity may indicate that calcium is also released from intracellular stores. This is in contrast to a previous report [28]. However, there was a change in the shape of the transients from narrow peaks in a calcium-free medium to broad and long-lasting calcium increases in calcium-depleted cells. This must be due to influx of calcium through yet uncharacterized calcium channels. Addition of thapsigargin decreased the transient activity, and the basal level of cytosolic calcium decreased gradually with time supporting the view that gradual store depletion invalidates the fine-tuning of the resting calcium concentration in the cytosol.

#### 4.1.2. Effect of Thapsigargin on Differentiating Neurons

Thapsigargin specifically inhibits the endoplasmic calcium ATPase activity thus inhibiting the uptake of calcium into the endoplasmic reticulum [42]. Thapsigargin (1 *μ*M) has been shown to induce a substantial depletion of intracellular Ca^2+^ stores in a HeLa cell derivative [43]. In order to examine if differentiating human neural stem cells contain intracellular stores of calcium supplied with calcium pumps, cells were incubated with 2 *μ*M thapsigargin. The observed transient increase of cytosolic calcium (Figure 3(a)) seemed to indicate an inhibition of a thapsigargin-sensitive pump normally counteracting calcium leak from intracellular stores. Increase of cytosolic calcium caused by influx of extracellular calcium was not a likely explanation since the cells were incubated in calcium-free solution with 10 *μ*M EGTA.

#### 4.1.3. Store-Regulated Calcium Influx in Differentiating Neurons

The concept of receptor-operated calcium entry was introduced more than 30 years ago [44]. It appeared to be a calcium-activated calcium release channel [45, 46]. Recently, a likely sensor for the luminal calcium concentration in the endoplasmic reticulum has been identified [47–49], and a second protein, Orai1 or CRACM, was identified as a modulator of CRAC (Ca^2+^ release-activated Ca^2+^ channels) or possibly the CRAC itself [50, 51]. We observed a rather fast cytosolic calcium increase by addition of calcium to differentiating stem cells primed with thapsigargin in a calcium-free medium, which is considered to empty the intracellular calcium stores. This is likely to reflect an opening of store-operated calcium channels in the cells, which are considered an important mechanism of calcium entry into the cytosol [52]. However, we cannot exclude the possibility that the increased cytosolic calcium may result from opening of voltage-gated calcium channels. Recently, exposure of a neural stem cell line (hVM1) for 60 mM potassium after 12 days of differentiation was shown to increase intracellular calcium in 9.5% of the cells, indicative of the development of excitability and calcium channels in the plasma membrane of these cells [53].

#### 4.1.4. Effect of Ryanodine on Differentiating Neurons

Ryanodine binds to CRAC in the sarcoplasmic reticulum of cardiac and skeletal muscles as well as to the endoplasmic reticulum of various tissues [54, 55]. These organelles constitute a major intracellular store of calcium in mammalian cells. Micromolar concentrations of ryanodine binds to a high-affinity site on the channels [56, 57] and locks the channel in a subconductance state with an open probability of unity [58–60]. Our observation of an increased number of calcium transients upon ryanodine treatment is in accordance with previous observations in rat chromaffin cells [61]. The time-dependent and gradual increase of cytosolic calcium in the presence of ryanodine may be explained by the development of the subconductance state of a ryanodine-sensitive calcium channel allowing the release of calcium to the cytosol. It may be speculated that the significant increase of the fraction of cells with calcium transients may be due to calcium-activated gating of channels in the plasma membrane or in the endoplasmic reticulum.

#### 4.1.5. Na^+^/Ca^2+^ Exchanger in Differentiating Neurons

Maintenance of low-cytosolic calcium concentrations requires effective transport mechanisms for removal of calcium into the intracellular stores and across the plasma membrane to the extracellular medium. The plasma membrane Ca^2+^-ATPase (PMCA) pumps, and Na^+^/Ca^2+^ exchangers extrude Ca^2+^ to the outside, whereas the endoplasmic reticulum ATPase (SERCA) pumps sequester Ca^2+^ to the intracellular stores [2]. The turnover rate of the Na^+^/Ca^2+^ exchangers is considerable higher than that of the Ca^2+^-ATPase (PMCA) pumps while the affinity for Ca^2+^ is lower [62] indicating that Na^+^/Ca^2+^ exchanger activity is important for the regulation of cytosolic Ca^2+^. Our observation of a time-dependent increase of cytosolic calcium in cells incubated in the absence of sodium may indicate a reduction of Ca^2+^ extrusion capacity and loss of homeostasis. The cells were incubated in a Ca^2+^-free solution for 10 min before the addition of calcium to the medium. This may contribute to lowering of the intracellular calcium stores and thus activation of the store-regulated calcium channels that allow influx of calcium upon the addition of calcium. Control experiments performed in the presence of sodium demonstrate that the effect of the calcium-free exposure was an increase of cytosolic calcium but to a lesser extent than that observed in a sodium-free medium. In accord, the AUC of the cells in a sodium-free medium was significantly larger than AUC from cells incubated in presence of sodium. We conclude that the plasma membrane of these cells contains an exchange mechanism for Na^+^ and Ca^2+^. It may be speculated that the increase of the fraction of cells with calcium transients in sodium-free solution results from opening of store-regulated calcium channels.

#### 4.1.6. Calcium Transients Induced by Carbacholine in Differentiating Neurons

Activation of muscarinic receptors M_1_, M_3_, and M_5_ is coupled to intracellular synthesis of inositol triphosphate (IP_3_), which open calcium channels in the endoplasmic reticulum and allowing efflux of calcium into the cytosol [63, 64]. We used the stable muscarinic agonist carbachol to study if the differentiating neurons responded to muscarinic activation [41] and then if the activation was transduced to intracellular release of calcium to the cytosol. The dose-dependent increase of the fraction of cells that respond to carbachol stimulation may indicate muscarinic receptor activation. This was supported by the observed inhibition by pirenzepine, which is considered a muscarinic M_1_ receptor antagonist [41]. In accord, quantifying the calcium transients (AUC) demonstrates both the dose-dependence increase of the response to carbachol and the inhibition by pirenzepine. It may thus be reasoned that carbachol activates an M_1_ muscarinic receptor in the PM of differentiating neural stem cells. Control experiments in Ca^2+^-free solution support the idea that the increased cytosolic Ca^2+^ is due to IP_3_-sensitive channels in the endoplasmic reticulum allowing efflux of Ca^2+^ into the cytosol. Furthermore, while the calcium-free solution did not influence the fraction of cells with calcium transients, it influences the quantitative parameter (AUC) of the cytosolic calcium increase. A possible explanation could be that coordinated calcium supply from intracellular stores and the extracellular medium is a requirement for large and long-term increase of cytosolic calcium.

#### 4.1.7. Possible Roles of Spontaneous Calcium Transients in Differentiating Neurons

We have previously investigated the effect of intracellular calcium transients on gene expression [65], which in immature cells might be a prerequisite for cell differentiation. Dolmetsch et al. [5] reported that intracellular calcium oscillations in T-lymphocytes increase both the efficacy and the information content of calcium signals that lead to gene expression and cell differentiation. Moreover, recent reports show that spontaneous Ca oscillations in human immature dendritic cells are linked to translocation of transcription factor NFAT (nuclear factor of activated T-cells) into the nucleus [66] and that endogenous calcium spike activity in *Xenopus tropicalis* regulates genetic pathways involved in neuronal development of transmitter specification [67]. We suggest that the spontaneous calcium transients that we have observed in our human midbrain-derived stem cells may contribute to mechanisms involved in their differentiation.

#### 4.1.8. Summary of Calcium Regulation in Differentiating Neurons

We have provided evidence for activity of intracellular calcium stores with sensitivity for thapsigargin and ryanodine, indicating the presence of endoplasmic reticulum ATPase (SERCA) pumps and ryanodine receptors. Furthermore, it is likely that our cells contain muscarinic M_1_ receptors in the plasma membrane that might initiate IP_3_ synthesis and thus activate IP_3_ receptors on the endoplasmic reticulum. Extrusion of calcium across the plasma membrane seems to occur by sodium-calcium exchange and influx of calcium by store-activated calcium channels. The spontaneous calcium transients are supplied by calcium from either the intracellular stores or the extracellular medium. Available calcium supply influences the pattern of activity. The quantitative largest calcium increase (AUC) seemed to require a coordinated calcium supply from both sources. Although it seems that these cells have developed the mechanisms necessary for control of calcium spiking as also reported for human mesenchymal stem cells, further experiments are necessary to evaluate the interplay between the mechanisms that lead to cytosolic calcium increase and decrease, respectively, as well as to elucidate a possible role of voltage-activated or receptor-activated calcium channels in the plasma membrane.

### 4.2. Proliferating Human Midbrain-Derived Stem Cells

#### 4.2.1. Evidence for Calcium Stores and Store-Regulated Ion Channels in the Plasma Membrane of Proliferating Stem Cells

Thapsigargin is known to block a calcium ATPase, which supplies energy to reuptake calcium to intracellular stores of calcium. The observed calcium transients in a major fraction of the cells (Supplemental Figure 3) support the view that the proliferating stem cells do contain intracellular calcium stores. The effect of thapsigargin may be explained by a disturbing effect of thapsigargin on the balance between release and reuptake of calcium into the stores resulting in depletion of the stores for calcium. Depletion of calcium stores is known to activate store-regulated calcium channels in the plasma membrane. When the cells were primed with thapsigargin in a calcium-free medium, the subsequent incubation with calcium (Supplemental Figure 4) induced a calcium transient which is likely to be the result of opening of calcium channels in the plasma membrane in response to the depletion of the intracellular stores. This view is supported by the results shown in Figure 6 that thapsigargin releases calcium from the stores and this causes an influx of calcium.

#### 4.2.2. Calcium Supply to Spontaneous Calcium Transients in Proliferating Stem Cells

We report spontaneous calcium transients in proliferating neural stem cells (Figures 7(a) and 7(b)). More than 55% of the cells displayed spontaneous transients during the observation period. The cells were loaded with Fluo-4 in the presence of calcium in order to avoid depletion of intracellular calcium. Then, the cells were incubated in the presence of calcium. Omission of extracellular calcium caused a significant decrease of the fraction of cells with calcium transients to about half the value of the control sample. This may indicate that extracellular calcium contributes to the intracellular calcium transients. However, in the absence of external supply of calcium, there is still a substantial fraction of cells with calcium transients, which is almost abolished when the cells were pretreated with thapsigargin in a calcium-free medium. This treatment is likely to empty intracellular calcium stores, suggesting that these could be the calcium supplier for the spontaneous calcium transients.

We conclude that there are spontaneous calcium transients in neural stem cells during proliferation and that the calcium supply for the transients is mediated via both intracellular stores and calcium influx from the extracellular medium.

#### 4.2.3. Calcium Transients in Proliferating Cells Induced by Carbachol

If the cells contain intracellular calcium stores, it is likely that they may respond to activation of plasma membrane receptors that produce inositol triphosphate. We have stimulated the cells with the acetylcholine analogue carbachol in a calcium-free medium and observed an increase of the fraction of cells with calcium transients compared to cells incubated in a calcium-free medium (*p* < 0.01). This effect was counteracted by pirenzepine, which is a selective inhibitor of M_1_ acetylcholine receptors. These observations support the view that proliferating neural stem cells contain intracellular calcium stores and a muscarinic M_1_ receptor in the plasma membrane. It is likely that carbachol activation induces synthesis of inositol triphosphate that releases calcium from the intracellular stores.

#### 4.2.4. Existence of a Na^+^/Ca^2+^ Exchanger in Proliferating Stem Cells

The decrease of intracellular calcium following the peak value of the transients induced by thapsigargin (Supplemental Figure 3) could be due to removal of cytosolic calcium through efflux across the plasma membrane since reuptake to intracellular calcium stores is unlikely in the presence of thapsigargin. The mechanism of extrusion could be an exchange of intracellular calcium for extracellular sodium. We have incubated stem cells in the absence of sodium since in a sodium-free medium, the extrusion of calcium is abolished. This induced a significant increase of the fraction of cells with calcium transients compared to the control value (*p* < 0.001) as well as of the baseline calcium content. Thus, our observation supports the view that proliferating neural stem cells have developed a Na^+^/Ca^2+^ antiporter in the plasma membrane.

## 5. Conclusion

This is the first comparative report of spontaneous calcium transients in proliferating and differentiating human midbrain-derived stem cells that provides evidence for mechanisms that are likely to be involved. We propose that the observed spontaneous calcium transients may influence signaling pathways involved in stem cell proliferation, phenotypic differentiation, and maturation.

## Supplementary Material

Supplementary Figure 1 Video of differentiating stem cells. Recordings through 5 min with a frequency of 0.33Hz of fluo-4-loaded differentiating human midbrain-derived stem cells using a laser-scanning microscope (Olympus FluoView FV1000MPE multiphoton laser scanning microscope). Supplementary Figure 2 Calcium transients induced by carbachol in differentiating stem cells. The differentiating cells were loaded with Fura-2 and then exposed to various concentrations of carbachol for 15 min in presence or absence of extracellular calcium and pirenzepine. (A) shows the dose-dependent increase of the fraction of cells with calcium transients in response to carbachol. Mean values and SEM are shown for cells incubated in presence (square) or absence (triangle) of calcium and in the presence of pirenzepine and calcium (circle). The values from two (carbachol 100 μM) and 3-8 independent experiments are shown. (B) shows the effect of increasing concentrations of carbachol on the total calcium increase (AUC) during the 15 min of exposure to carbachol. Mean values and SEM from 43-194 cells. Supplementary Figure 3 Thapsigargin induced calcium transient in proliferating stem cells suspended in calcium-free medium. Cells were loaded in a calcium containing medium, and then washed in a medium containing calcium. Incubation was performed in a calcium-free medium with 10 μM EGTA. Thapsigargin (2 μM, final concentration) was added to the cells during the incubation. The figure shows the results from two independent experiments. Abscissa: Time of incubation (sec). Ordinate: Intracellular calcium content. The fluorescence intensity was normalized to the level of the fluorescence intensity of untreated cells (100 %). Mean values and S.E.M. from 50 and 39 cells from two independent experiments, responding to the addition of thapsigargin. The cells represent 76% and 60% of the observed cell population, respectively. Supplementary Figure 4 Calcium-induced calcium increase in calcium-depleted proliferating stem cells. The cells were calcium-depleted by preincubation with 2μM thapsigargin for 30 min in calcium-free medium containing 10μM EGTA, washed and incubated in a calcium-free medium with EGTA but without thapsigargin. Calcium (2mM, final concentration) was added to the cells after 200 sec incubation (arrow). Time course of calcium increase from a representative experiment (A) and the mean value of the peak levels from 7 independent experiments (B). Mean and S.E.M. from 81 cells (A) and from seven independent experiments (B). Taken together 332 cells were studied in seven experiments, and all the cells responded to the addition of calcium.
