# Supplementary figures and images for: Comparative Analysis of Spontaneous and Stimulus-Evoked Calcium Transients in Proliferating and Differentiating Human Midbrain-Derived Stem Cells
Source: Stem Cells Int. 2017 Oct 22;2017:9605432. doi: 10.1155/2017/9605432 (PMC5671755; doi:10.1155/2017/9605432)

## Slide 1
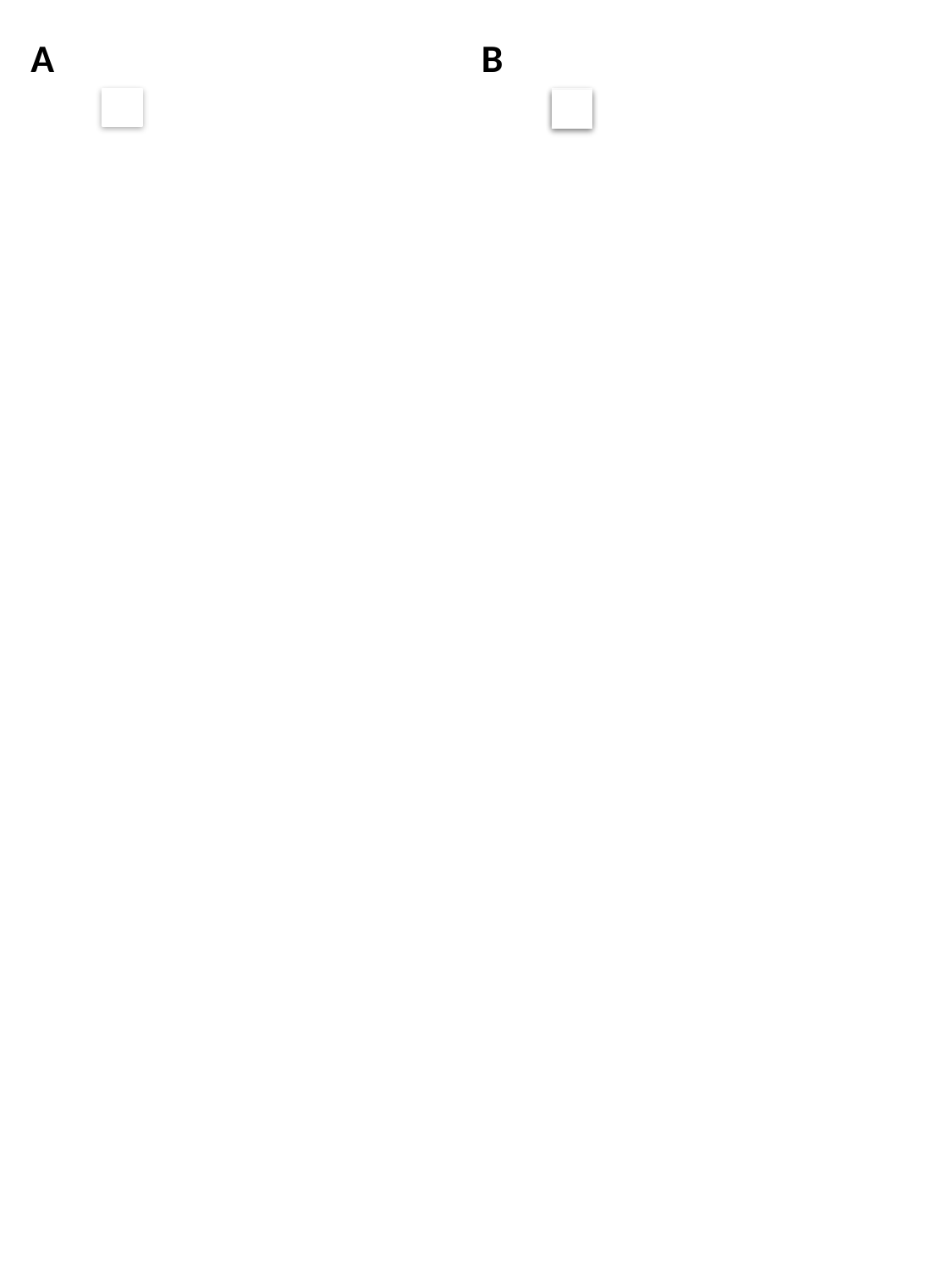

A
B

Supplement: Supplementary file 2 [file 9605432.f2.pptx]

## Slide 1
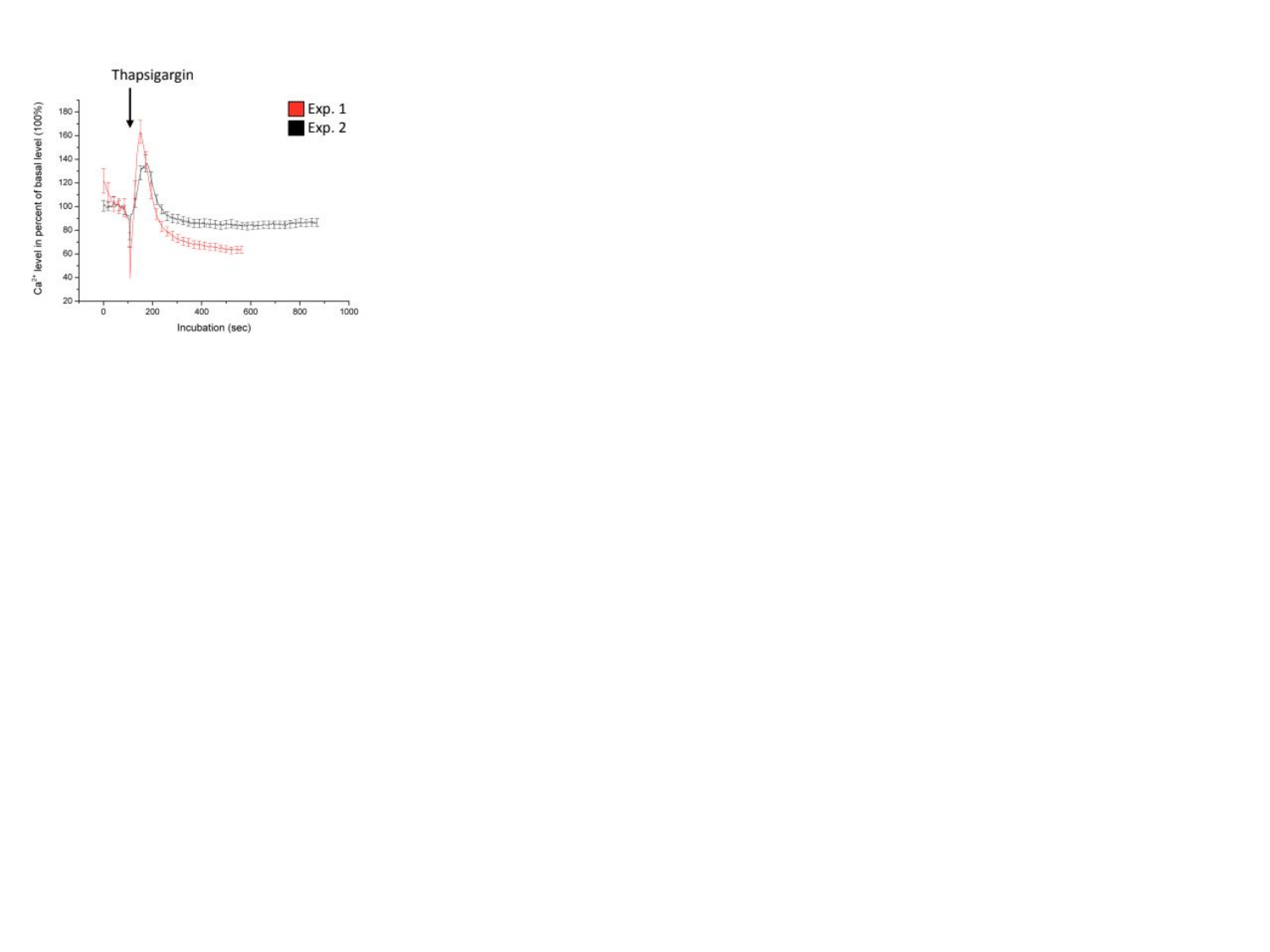

Supplement: Supplementary file 3 [file 9605432.f3.pptx]

## Slide 1
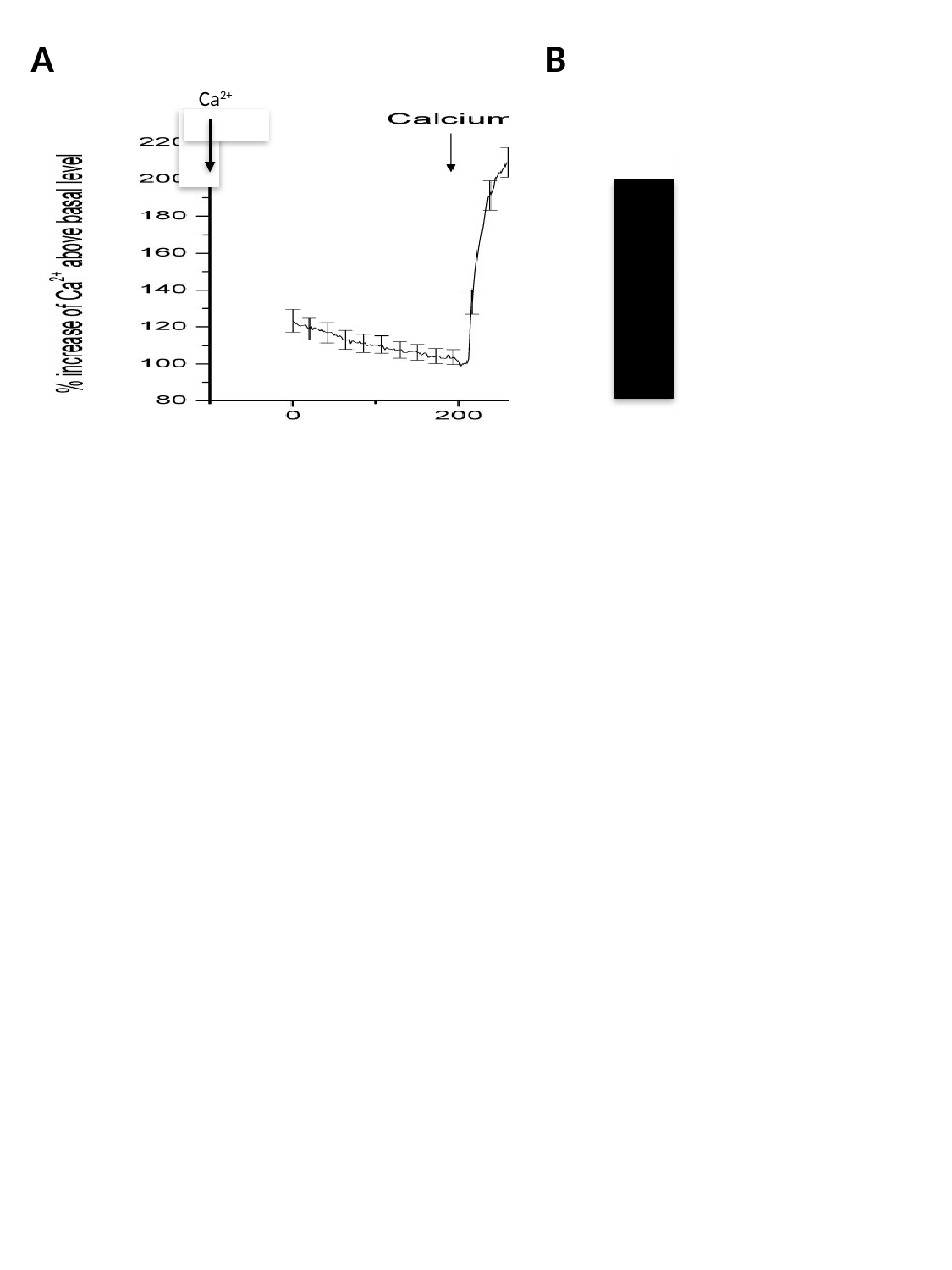

A
B
Ca2+
Exp. 1
Exp. 2

Supplement: Supplementary file 4 [file 9605432.f4.pptx]
